# Pigmented Basal Cell Carcinoma Masquerading as a Melanoma

**DOI:** 10.7759/cureus.4369

**Published:** 2019-04-02

**Authors:** Boya Abudu, Philip R Cohen

**Affiliations:** 1 Internal Medicine, Kaiser Permanente Oakland Medical Center, Oakland, USA; 2 Dermatology, San Diego Family Dermatology, National City, USA

**Keywords:** basal, carcinoma, cell, collision, masquerading, melanoma, mimic, nodular, pigment, pigmented

## Abstract

Basal cell carcinoma is the most common skin cancer. Pigmented basal cell carcinoma is an uncommon clinical presentation that can resemble a melanoma. We present the clinical and pathologic features of three individuals whose pigmented basal cell carcinomas masqueraded as melanomas. All of the patients were Hispanic and ranged in age from 63 years to 77 years. They presented with a pigmented lesion that was ultimately diagnosed as a pigmented basal cell carcinoma; one woman had a collision tumor consisting of a pigmented basal cell carcinoma and a seborrheic keratosis. All of the patients had their tumors removed using Mohs micrographic surgery, without recurrence. The clinical differential diagnosis of a black tumor―particularly in patients with darker skin types―should include pigmented basal cell carcinoma in addition to melanoma; a biopsy of the lesion will establish the diagnosis.

## Introduction

Common cutaneous malignancies include basal cell carcinoma, squamous cell carcinoma, and malignant melanoma [1-3]. Basal cell carcinoma is the most common skin cancer; a less common clinical presentation occurs when the tumor is pigmented [4-5]. In some circumstances, pigmented basal cell carcinoma can morphologically mimic a melanoma [6]. We present three individuals whose pigmented basal cell carcinoma masqueraded as nodular melanomas.

## Case presentation

Case 1

**Figure 1:**
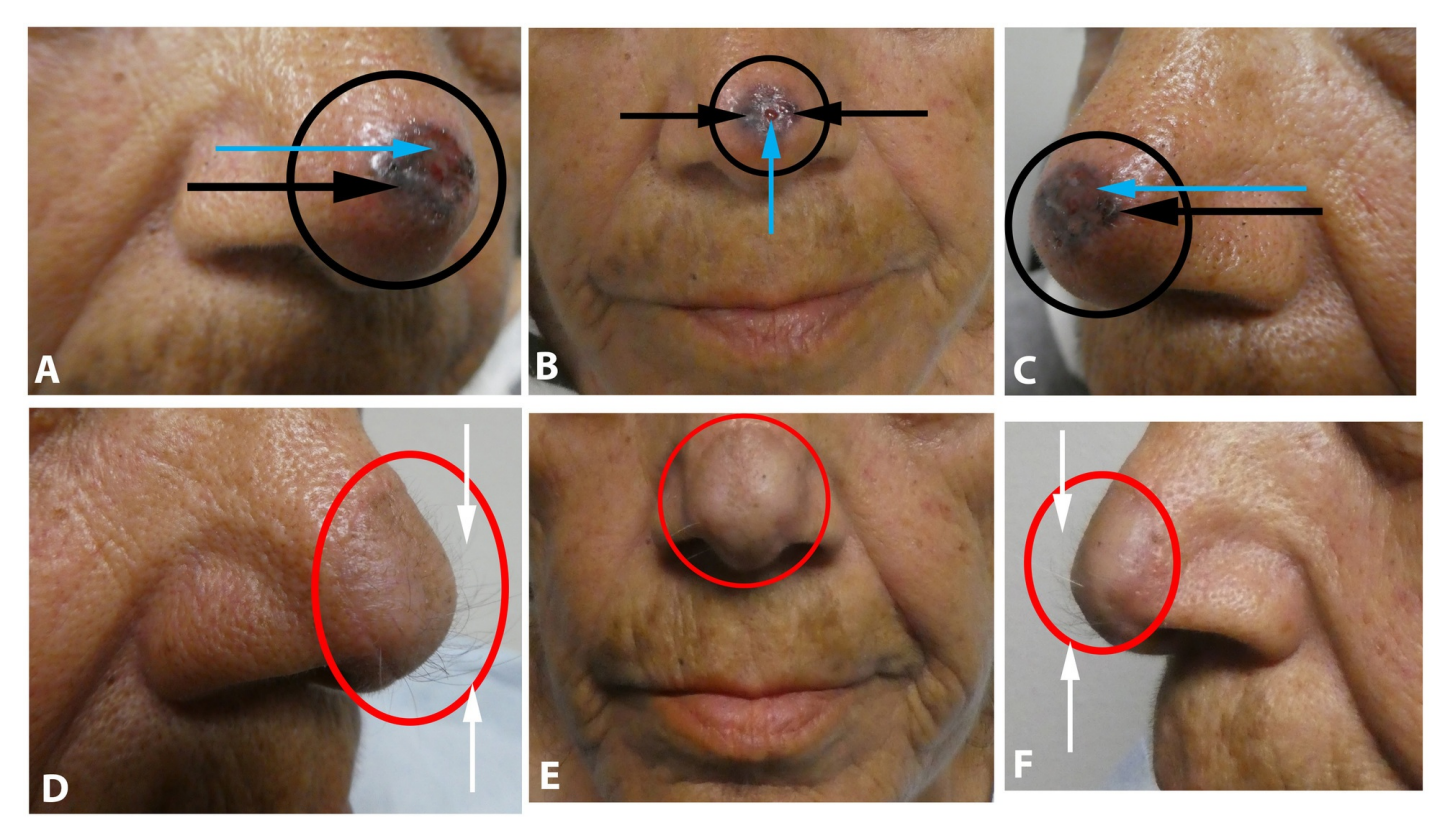
Pigmented basal cell carcinoma on the nasal tip mimicking melanomas Views of the basal cell carcinoma (within the black circle) prior to biopsy, from the right side (A), frontal (B), and left side (C), of the nasal tip of a 74-year-old Hispanic woman demonstrate an ulcerated (blue arrows), black nodule (black arrows). A paramedian forehead flap was used to repair the wound following Mohs micrographic surgery; the right side (D), frontal (E), and left side (F) of her nasal tip show excellent healing of the flap (within red circle) and hypertrichosis (white arrows) from the tissue used to cover the surgical defect.

A 74-year-old Hispanic woman presented with an asymptomatic lesion of one-year duration on the nasal tip. Clinical examination showed a nodular tumor; in addition to being black, there was ulceration (Figures 1A-1C). There was no palpable neck lymphadenopathy. Morphologically, the clinical differential diagnosis included malignant melanoma.

A biopsy was performed. Microscopic examination showed nodular aggregates of basaloid tumor cells extending from the epidermis into the dermis. There was pigment not only in the tumor cells but also within the melanophages in the adjacent dermis. Correlation of the clinical presentation and pathology established the diagnosis of pigmented nodular basal cell carcinoma.

Mohs surgery was performed. The tumor was cleared after three stages. A left paramedian forehead flap was performed to treat the surgical wound. Follow-up three months later showed excellent healing without recurrence of the skin cancer. However, there was significant hypertrichosis involving the tissue flap on the nasal tip (Figures 1D-1F). This was remedied by using electrolysis to eliminate the hair.

Case 2

**Figure 2:**
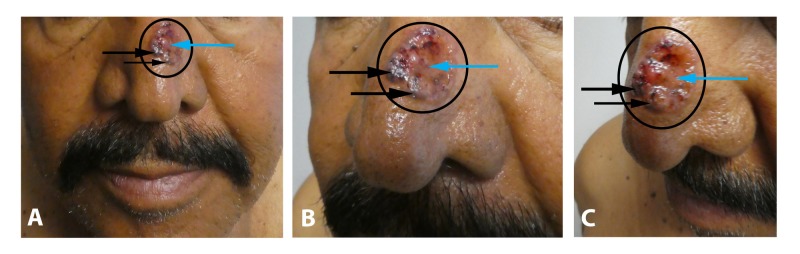
Pigmented basal cell carcinoma on the left nasal bridge mimicking melanoma Distant (A), closer (B and C), frontal (A and B), and left side (C) views of a pigmented basal cell carcinoma (within the black circle) on the left nasal bridge of a 63-year-old Hispanic man. The tumor consists of black pigment (black arrows) adjacent to ulcerated (blue arrow) and intact red and flesh-colored areas.

A 63-year-old Hispanic man presented with an asymptomatic lesion on his left nasal bridge of nine months duration. The tumor appeared as an ulcerated plaque with black pigmentation; there were also red and flesh-colored areas (Figure 2). There was no palpable neck lymphadenopathy. The clinical differential diagnosis included ulcerated malignant melanoma.

A biopsy was performed. Microscopic examination showed nodular aggregates of basaloid tumor cells extending from the epidermis into the dermis. There was pigment not only in the tumor cells but also within the melanophages in the adjacent dermis. Correlation of the clinical presentation and pathology established the diagnosis of pigmented nodular basal cell carcinoma.

Mohs surgery was performed. The tumor was cleared in two stages. A full thickness graft was used to treat the surgical wound. Follow-up after three months did not reveal any recurrence of the cancer.

Case 3

**Figure 3:**
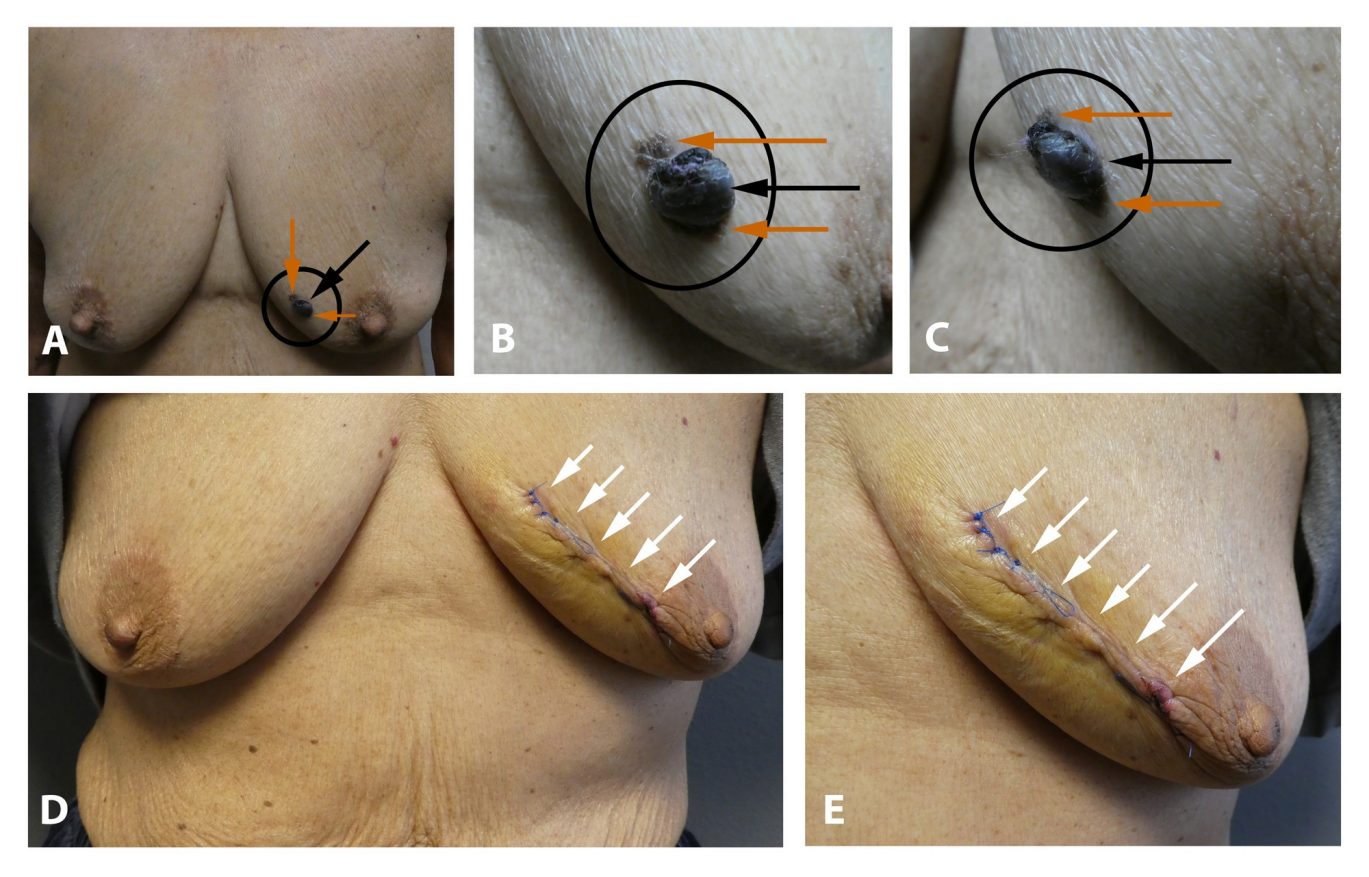
Pigmented basal cell carcinoma (with concurrent seborrheic keratosis) masquerading as melanoma. Distant (A) and closer (B and C) views of a collision tumor consisting of a pigmented basal cell carcinoma that presented as a large ulcerated black nodule (black arrow) and a seborrheic keratosis (orange arrows) appearing as pigmented patches adjacent to the centrally located black nodule on the left breast of a 77-year-old woman. Distant (D) and closer (E) views of the left breast following a layered closure (white arrows) of the surgical wound. The tumor was cleared after one stage of Mohs micrographic surgery.

A 77-year-old Hispanic woman presented with a lesion of one-year duration that was progressively enlarging on her left breast; the lesion would occasionally bleed. Clinical examination showed a 2 x 1 cm black nodule; in addition, extending from the base of the tumor onto the adjacent skin, was macular brown pigmentation (Figures 3A-3C). There was no palpable neck, axillary, or inguinal lymphadenopathy. The clinical differential diagnosis included a nodular malignant melanoma.

A biopsy was performed. Microscopic examination showed that the lesion consisted of two concurrent tumors. The first was an ulcerated nodular basal cell carcinoma with aggregates of basaloid tumor cells extending from the epidermis into the dermis; there was pigment in both the tumor cells and within the melanophages in the adjacent dermis. The second was a seborrheic keratosis showing acanthosis and hyperpigmentation. Correlation of the clinical presentation and pathology established the diagnosis of a collision tumor consisting of an ulcerated nodular basal cell carcinoma and a seborrheic keratosis.

Mohs surgery was performed. The tumor was cleared in one stage. A layered closure was used to repair the surgical defect (Figures 3D-3E). Follow-up after three months did not reveal any recurrence.

## Discussion

Clinical presentations of basal cell carcinoma include nodular basal cell carcinoma, superficial basal cell carcinoma, morpheaform basal cell carcinoma, red dot basal cell carcinoma, and pigmented basal cell carcinoma [7-9]. Pigmented basal cell carcinoma is an uncommon morphologic presentation [10]. Maloney et al. found that among a series of 1,039 consecutive basal cell carcinomas, only 70 (6.7%) were pigmented [11]. Pigmented basal cell carcinoma is usually seen in skin of color, including black, Hispanic, and Asian individuals [12-13]. They occur less frequently in Caucasians.

The clinical and pathologic features of our patients are summarized in Table 1. Basal cell carcinomas usually occur in older individuals [14-15]. Our patients ranged in age from 63 years to 77 years and the median age was 74 years.

**Table 1:** Features of three patients whose pigmented basal cell carcinoma mimicked melanoma. Abbreviations: BCC-P, basal cell carcinoma – pigmented: pigmented basaloid tumor nodules and dermal melanophages; C, case; FTSG, full-thickness skin graft; L, left; LC, layered closure; M, man; MMS, Mohs micrographic surgery; PMFF, paramedian forehead flap; SK, seborrheic keratosis: acanthosis and hyperkeratosis; W, woman

C	Age	Ethnicity	Gender	Location	Appearance	Pathology	Treatment
1	74 years	Hispanic	W	Nasal tip	Black nodule with ulceration	BCC-P	MMS and PMFF
2	63 years	Hispanic	M	L nasal bridge	Black nodules; red and flesh-colored areas; ulceration	BCC-P	MMS and FTSG
3	77 years	Hispanic	W	L medial breast	Ulcerated black nodule; brown patch extending from the tumor base onto the adjacent skin	Collision tumor: BCC-P and SK	MMS and LC

All of our patients were Hispanic and had darker skin. Two of the patients had their basal cell carcinoma on a sun-exposed area―their nose. However, one woman had a large tumor on her left breast, an area that received no prior sun exposure.

Pigmented basal cell carcinoma presents as black papules or nodules. Its morphologic appearance can mimic that of a melanoma [16]. Therefore, a biopsy is necessary to establish the diagnosis of a pigmented basal cell carcinoma and exclude the possibility of melanoma.

Microscopic findings of a pigmented basal cell carcinoma are often those observed in a nodular basal cell carcinoma: nodular aggregates of basaloid tumor cells extending from the epidermis into the underlying dermis. There is often peripheral palisading of the cells at the edge of the tumor nests [17-18]. However, in addition, pigmented basal cell carcinomas harbor pigment present within the tumor cells, in melanophages in the adjacent dermis, or both; this accounts for the black appearance of the tumor [19].

One of our patients had a collision tumor [20]. This phenomenon occurs when two different lesions concurrently occupy the same location. The seborrheic keratosis that extended from the margins of the basal cell carcinoma mimicked the hyperpigmentation that can occasionally be found in the epithelium immediately adjacent to a melanoma.

The management of a pigmented basal cell carcinoma is the same as that of a non-pigmented basal cell carcinoma. Excision of the residual tumor is often performed. For facial lesions, Mohs micrographic surgery is the treatment of choice.

## Conclusions

Pigmented basal cell carcinoma occurs more commonly in patients with skin of color and may clinically resemble melanoma. Thus, the differential diagnosis of a black nodule or ulcerated black plaque should include pigmented basal cell carcinoma, in addition to melanoma. Similar to the patients in this report, a biopsy of the tumor will establish the diagnosis.
